# Ultraviolet protection of *Bacillus thuringiensis* through microencapsulation with Pickering emulsion method

**DOI:** 10.1038/s41598-020-77721-8

**Published:** 2020-11-26

**Authors:** Elham Jalali, Shahab Maghsoudi, Ebrahim Noroozian

**Affiliations:** 1 Department of Chemistry, Shahid Bahonar University of Kerman, P.O. Box 76169-133, Kerman, Iran; 2 Young Researchers Society, Shahid Bahonar University of Kerman, P.O. Box 76175-133, Kerman, Iran

**Keywords:** Biological techniques, Biotechnology, Chemical biology, Nanoscience and technology

## Abstract

An encapsulated formulation of *Bacillus thuringiensis* (*Bt*) was produced by the Pickering emulsion technique to improve its activity and stability under UV-A radiation. In this technique latex particles, GO nanosheets, olive oil, ethanol, and water were used to encapsulate *Bt* in colloidosomes. The protective efficacy of this formulation in protecting *Bt subsp. Kurstaki* against deactivation by UV-A irradiation was measured, so that spore viability and mortality on *Ephestia kuehniella* (*E. kuehniella*) *Zeller* larvae under UV-A radiation are investigated. According to the results of both tests, encapsulated formulation at a concentration of 0.045% has the highest protection of viability. Hence, colloidosome microcapsule formulations successfully provide good protection against UV radiation.

## Introduction

Microbial insecticides, due to their safety to humans and the environment, have been used in pest management^1^. *Bacillus thuringiensis* (*Bt*) is an aerobic Gram-positive soil bacterium^2^, commercial products of which are powders containing a mixture of dried toxin crystals and spores^3^. Today, there are over 400 registered *Bt*-based formulations containing insecticidal proteins and viable spores in the market. Lack of persistence to UV radiation is the main weakness of many formulations of *Bt*^4^. It is known, however, that the formulation of pesticides has overriding significance for optimization of its efficacy. This defect can be improved by developing better formulations of *Bt*. Here, we look for a formulation that has the most protective effect and at the same time has the least impact on non-target organisms^5^. Many different methods have been assayed as UV protectants to improve the activity of *Bt* in field environments. Microencapsulation has recently attracted substantial attention in protecting pathogens from UV light due to the probability of controlling the release rate of an effective agent^6^. It not only enhances the activity of control agents and the performance of the formulation against ultraviolet radiation, but also improves the slow release of the active component^7^. Microencapsulation of biopesticides based on living organisms (e.g. bacteria, fungi, viruses) has a defect that is often accompanied by the decline of activity^8,9^. In this work, we introduce a novel encapsulated formulation of *Bt* based on the fabrication of Pickering emulsions. The Pickering emulsion technique has become increasingly important as a template for capsule formation and relies on solid-stabilized emulsions^10^. The droplet is confined by connecting colloidal particles adsorbed to the emulsion droplets to a solid shell. The formed liquid core, solid-shell microcapsules are called colloidosomes^11^. Increased sturdiness and a capsule permeability controlled by the particle size are the benefits of colloidosomes^12^. If the capsules can maintain and protect the encapsulated material until delivery conditions are reached in the target environment, then the conditions are ideal^13,14^. The release could be caused by a change in the system conditions of the delivery medium, such as its salinity, temperature, or pH. The defined permeability of colloidosomes for encapsulated species can be tailored through variation of the dimensions of the colloidal particles. By changing the dimensions of the colloidal particles, one can adjust the permeability of colloidosomes for encapsulated species, which is dependent on the size of the interstitial pores between the particles in the shell^15–17^. The ability to vary the pore size of the colloidosomes makes them appropriate for a broad variety of applications^18^.

We focused our research on the use of nanotechnology by considering the unique properties of graphene oxide (GO) for applied applications. In this study, we examined the effect of colloidosomes formed using Pickering emulsions, in which *Bt* was successfully encapsulated in colloidosomes using latex particles, GO nanosheets, olive oil, ethanol, and water. A low-temperature technique was used for the fabrication of water-core colloidosomes^7^. The self-assembly of particles at the liquid–liquid interface is a beneficial method of forming encapsulation materials. Herein, among various materials and techniques that have been developed to fabricate colloidosomes, we report pH-responsive colloidosomes containing *Bt* crystals that were formed by Pickering emulsions and stabilized by GO nanosheets. The four advantages of these microcapsules include, the release of *Bt* crystals in the midgut of lepidopteran larvae, especially when pH > 8; formation of aqueous formulation which could be dispersed in water; prevention of light-induced damage of the *Bt* spore; and maintaining the bioactivity of *Bt.* The present work is aimed to evaluate the toxicity of the *Bt* on *Ephestia kuehienlla (E. kuehniella) Zeller* larvae through bioassay. The main objective was the development of biological agents to improve the protective effect of the GO nanosheets on the stability of formulations exposed to UV radiation. Therefore, the spore viability and the crystal activity of encapsulated formulations were measured.

## Results

### Structure and properties of GO

**Figure 1:**
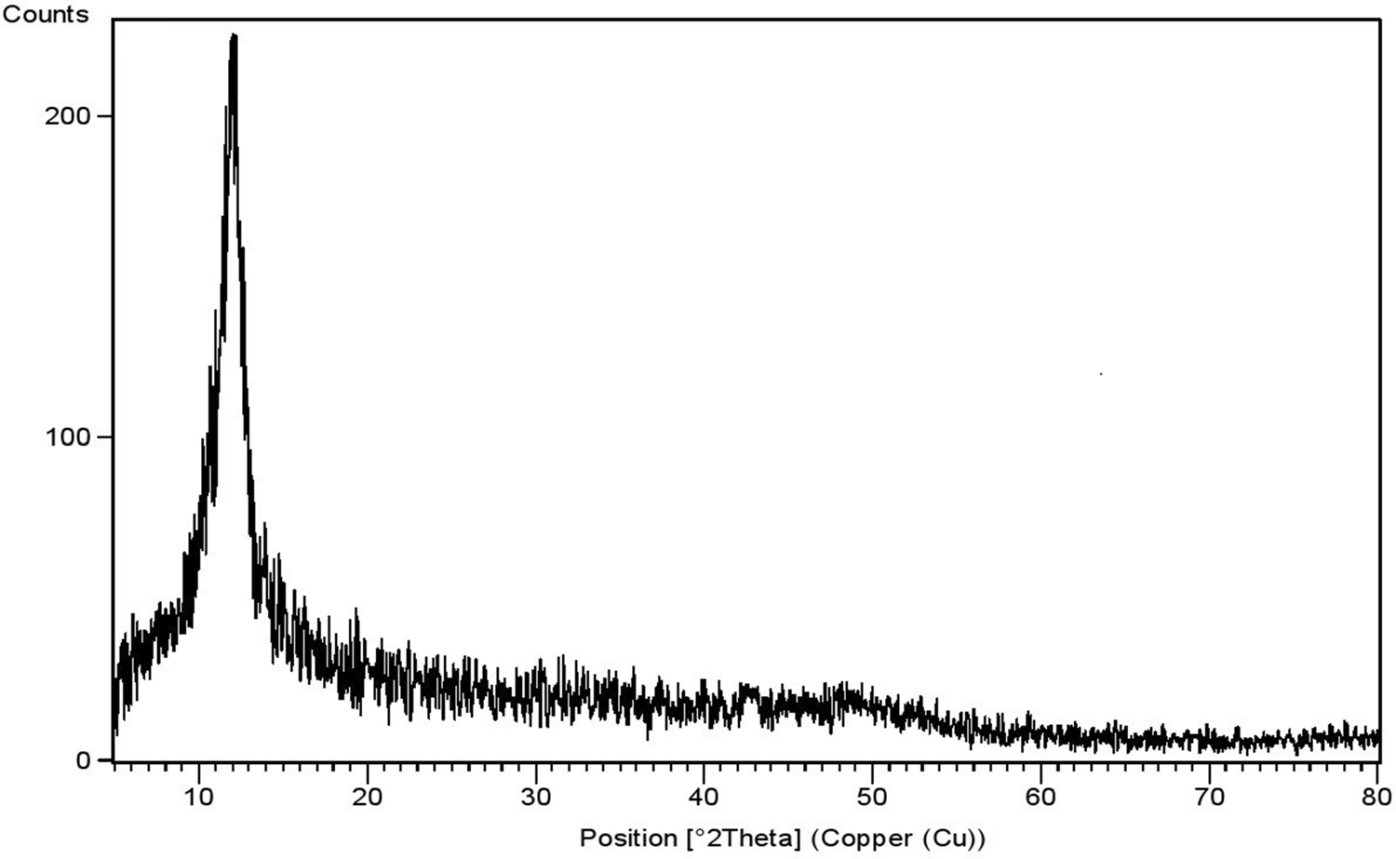
XRD pattern of GO nanosheets.

The XRD pattern of GO presented in Fig. 1 illustrates a sharp diffraction peak located at 2θ = 12.02°. The interlayer spacing of GO is 0.73 nm, which is more than the interlayer spacing of graphite (0.33 nm). Graphite has a sharp diffraction peak at 26.34°. The evidence confirms that oxygen-containing functional groups had been introduced to layers and successful oxidation of graphite into GO. An increased interlayer distance might be due to the formation of oxygen-containing functional groups, such as carboxyl, hydroxyl, and epoxy. The results of XRD proved the successful synthesis of the GO sheets by oxidizing graphite.

**Figure 2:**
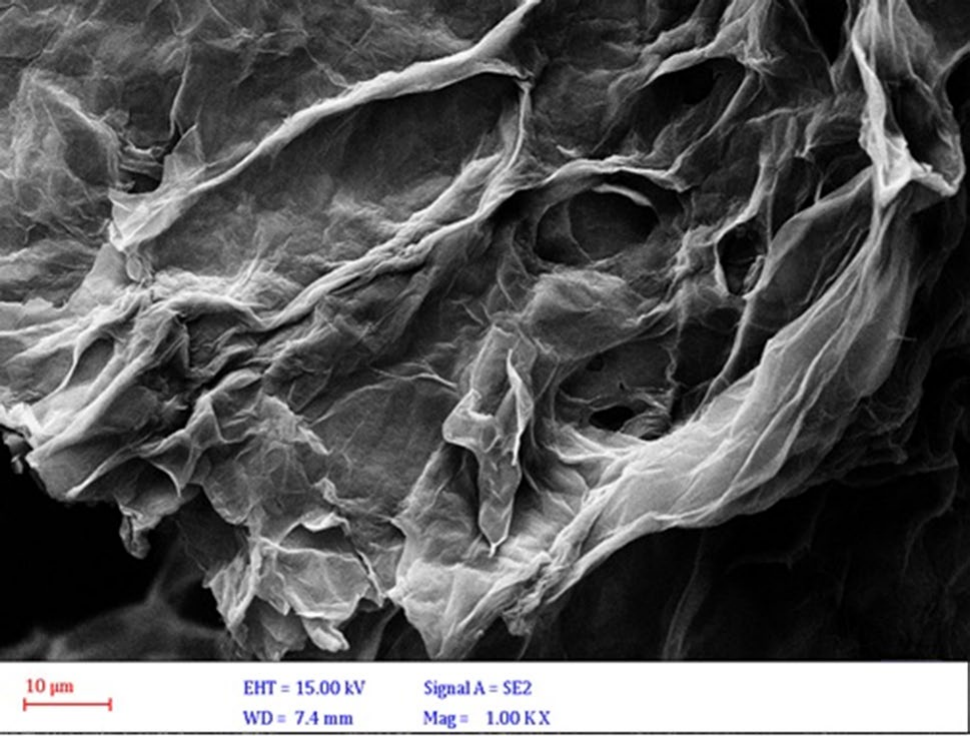
FESEM image of GO nanosheets.

The FESEM image of surface GO nanosheets is shown in Fig. 2. It was found that GO nanosheets consist of crumpled thin sheets and a wrinkled structure with an irregular surface. This result confirms the two-dimensional nanosheets of GO.

**Figure 3:**
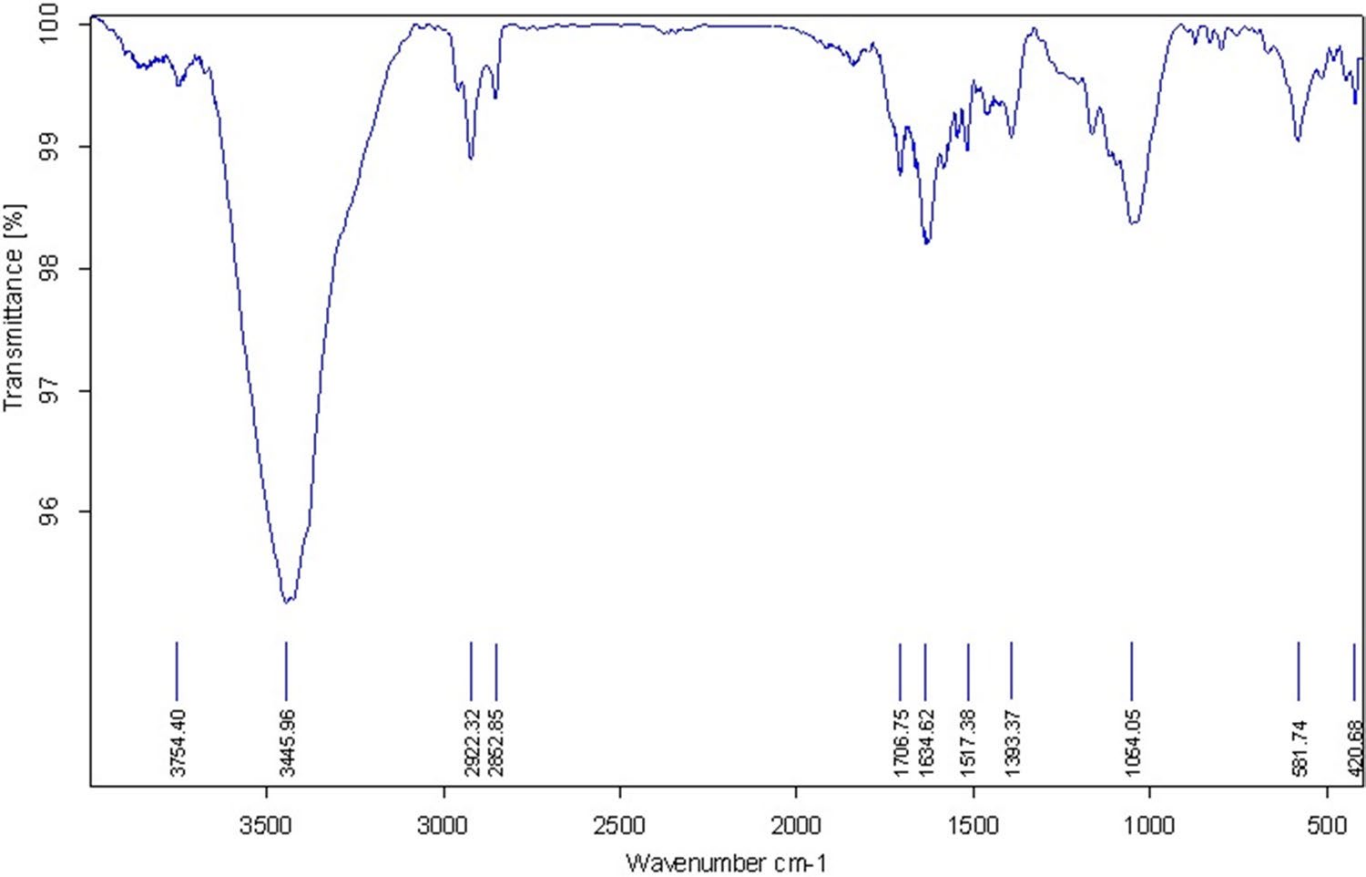
FTIR spectrum of GO nanosheets.

The chemical structure of GO nanosheets was investigated by FT-IR and the characteristic FTIR spectrum of GO nanosheets is depicted in Fig. 3. The characteristic peaks of 1054, 1393, 1634, 1706 and 3445 cm^−1^ corresponding to C–O, C–OH, C=C, C=O and O–H stretching vibrations, respectively confirm the formation of GO.

### Preparation of the microcapsule

**Figure 4:**
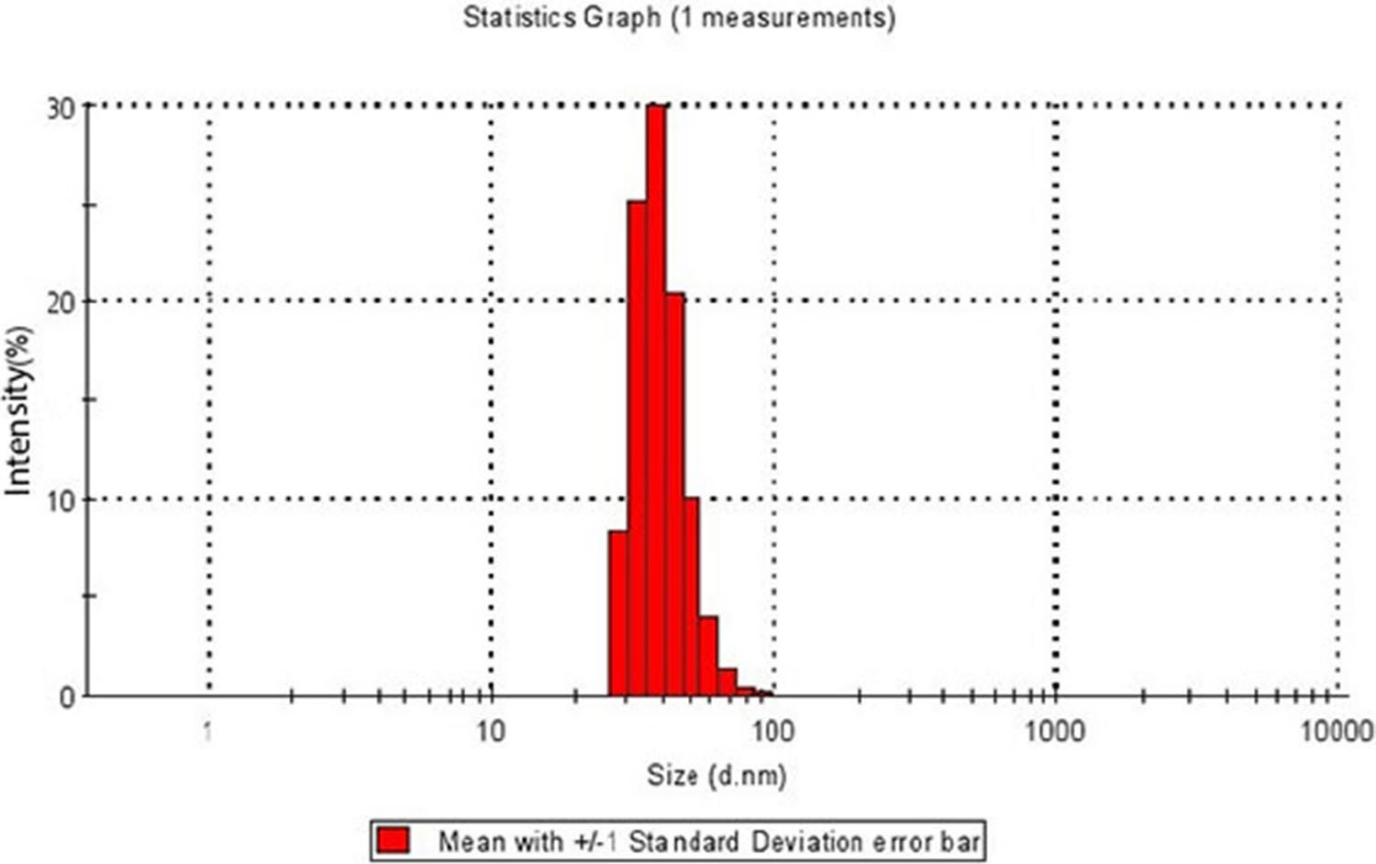
Size distribution chart of polymer latex particles measured using the DLS technique.

**Figure 5:**
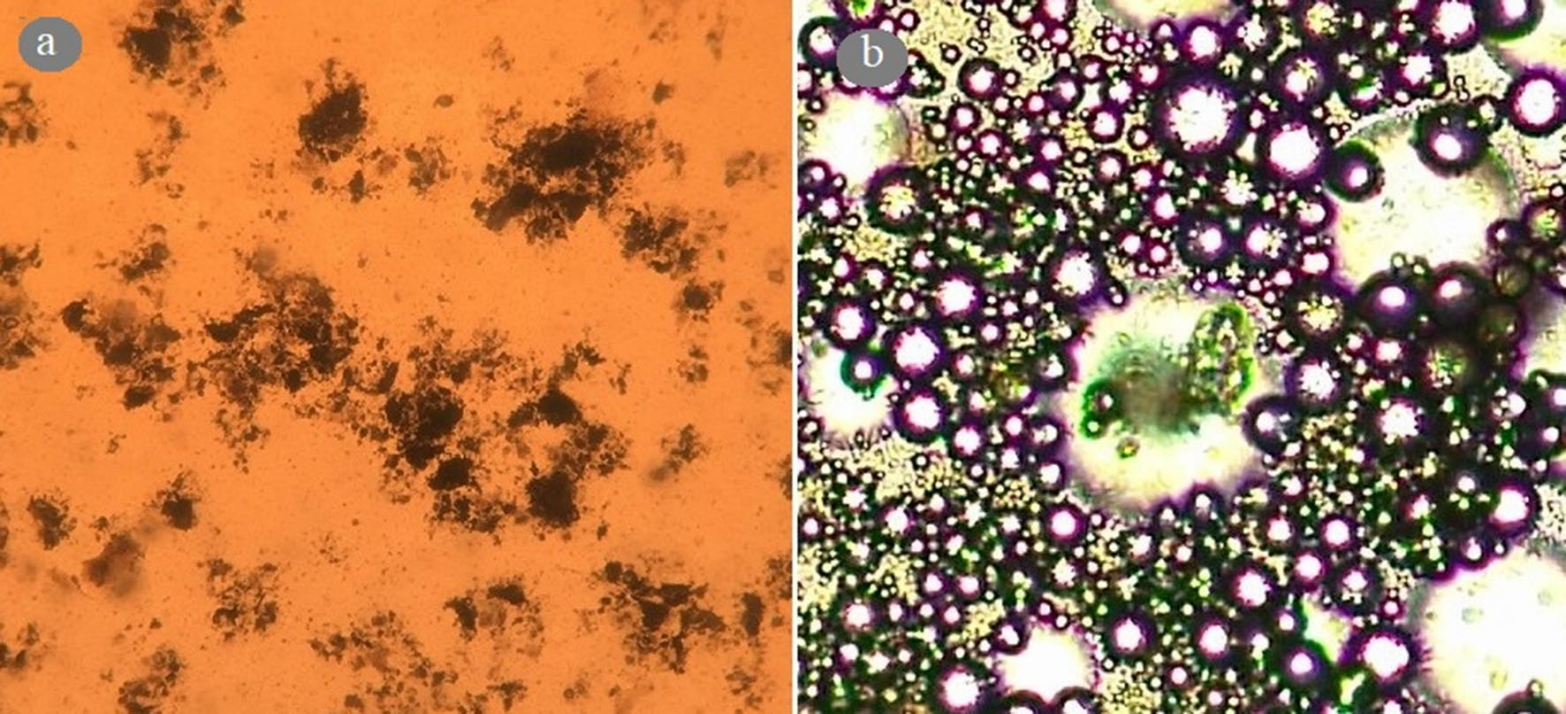
Optical microscope image of (**a**) mixture of *Bt* and polymer latex particles before the formation of colloidosomal microcapsules and (**b**) colloidosomal microcapsules containing the encapsulated *Bt* (40X).

**Figure 6:**
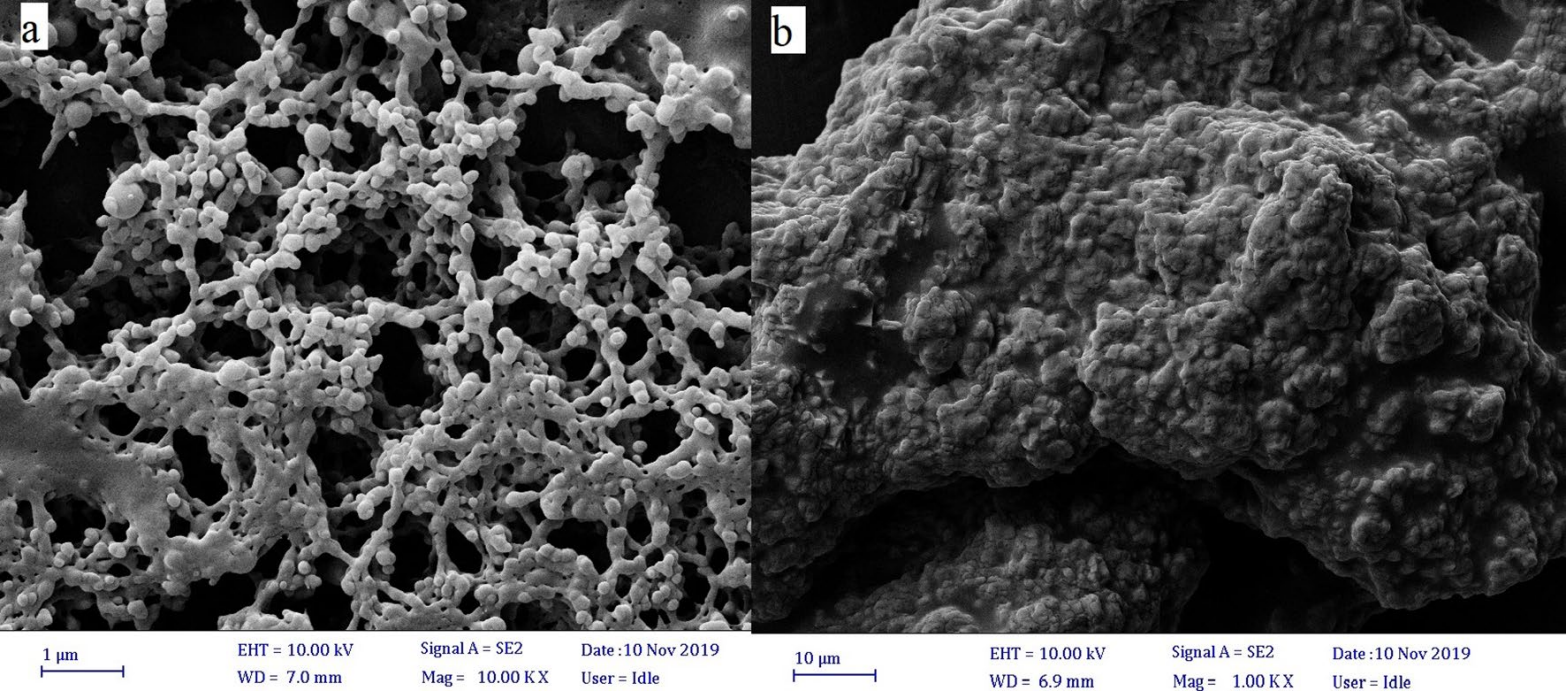
FESEM images of (**a**) polymer latex particles (**b**) colloidosomal microcapsules containing the encapsulated *Bt* after removal of the oil phase from a Pickering emulsion.

**Figure 7:**
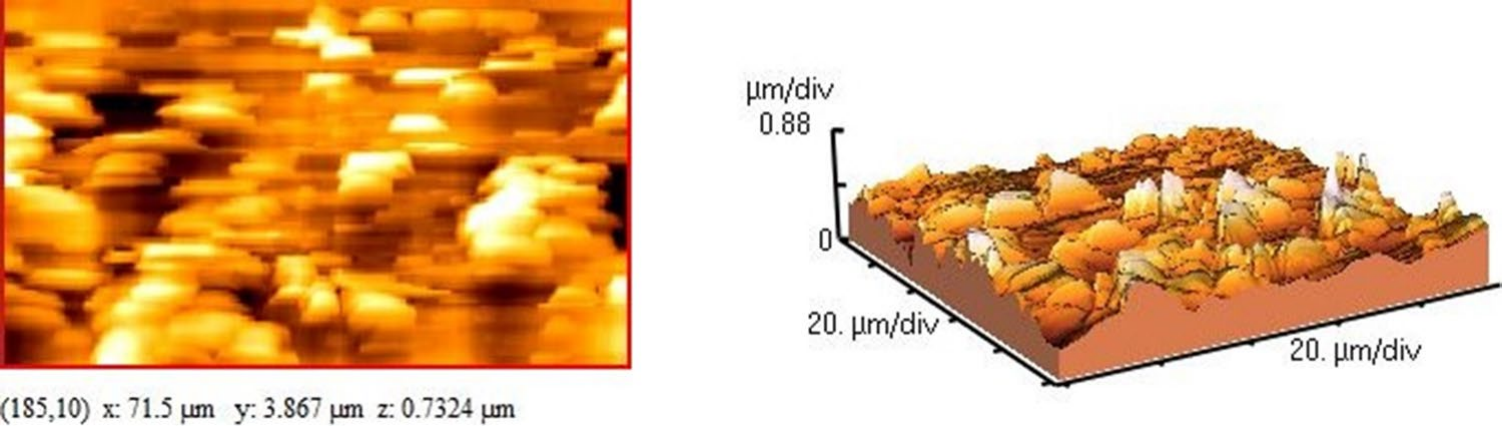
AFM images of colloidosomal microcapsules containing the encapsulated *Bt.*

**Figure 8:**
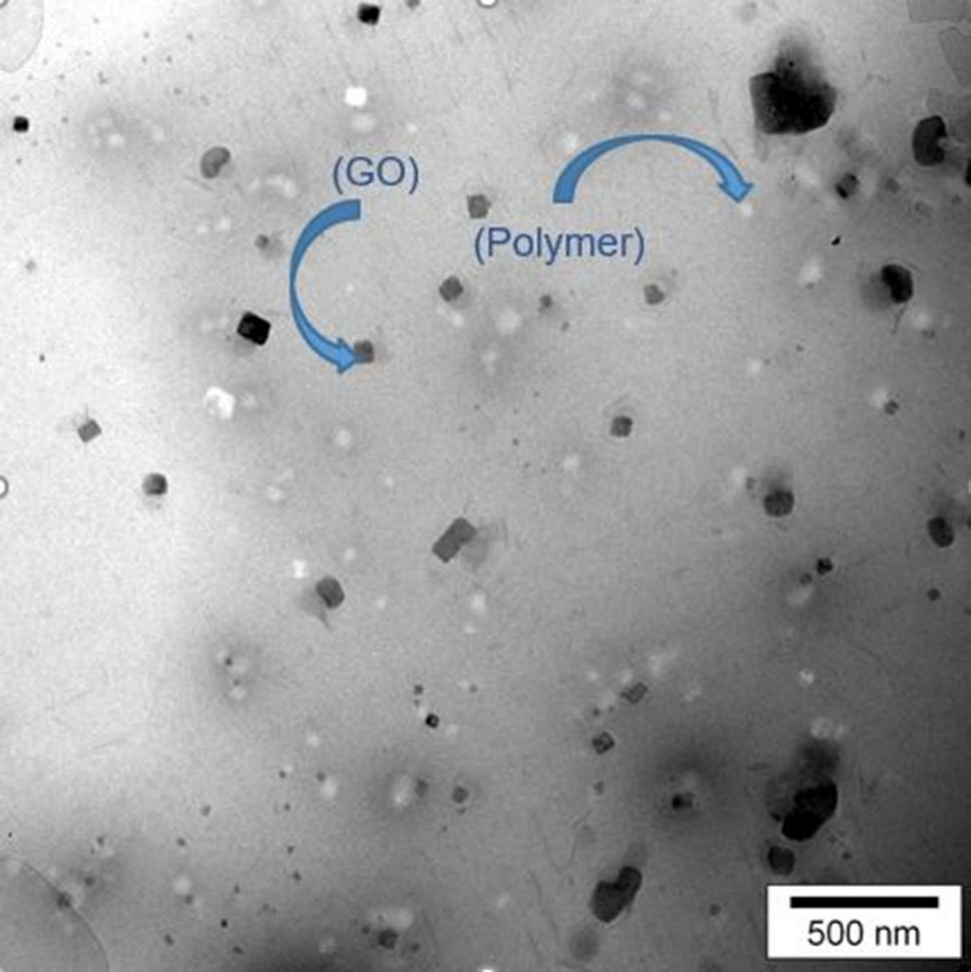
TEM image of colloidosomal microcapsules.

An emulsion polymerization process was applied for the preparation of colloidosomal microcapsules by a pH-sensitive latex of poly(methyl methacrylate-co-methacrylic acid). According to the DLS measurements, the average diameter of the polymer latex particles was 54.5 nm (Fig. 4). As shown in Fig. 4, the graph shows a narrow distribution range. Also, polymer latex particles before the formation of colloidosomal microcapsules and after their formation were characterized by optical microscopy (Fig. 5a,b). The water-core emulsions were found to have a hollow structure. Figure 6a shows the FESEM images of a mixture of *Bt* and polymer latex particles. Figure 6b reveals the FESEM images of colloidosomal microcapsules containing the encapsulated *Bt*. These results indicate the hollow structure of latex particles and that the aggregation of the latex particles causes the stabilization of the water-core colloidosomes. The water-core emulsions are stabilized by interfacial nanosheets. These nanosheets could stabilize the emulsion in the colloidal sense. They eventually lead to phase separation with the prevention of water droplets fusion. Figure 7 shows the AFM image of colloidosomal microcapsules containing the encapsulated *Bt*. R_ms_ and R_a_ values were obtained as 135.7 and 96.91 nm, respectively. As shown in Fig. 8, TEM image shows two different nanostructures that confirm the presence of GO nanosheets and polymer latex in the microcapsule.

### Assessment of the persistence of the microencapsulated formulations of *Bt* against UV-A

Evaluation of the persistence of microencapsulated formulation of *Bt* revealed that, after 96 h of exposure to UV radiation, spore count for non-protected *Bt* and 0.009%, 0.018%, 0.045%, and 0.09% GO encapsulated formulations declined to 9.68 × 10^8^, 15.67 × 10^8^, 16.13 × 10^8^, 17.56 × 10^8^, 15.11 × 10^8^, respectively (Table 1).

**Table 1:** The spore viability%, larval mortality%, and spore count for the microencapsulated formulation of at different concentrations of GO against UV-A.

Treatment	Spore viability %	Larval Mortality %	cfu (10^8^ spores/mL)
Non-irradiated non protected *Bt*	100 ± 0.00^e^	100 ± 0.00^f^.	32 ± 1.19^d^
Irradiated non protected *Bt*	30.27 ± 0.43^a^	38.20 ± 0.49^a^	9.68 ± 0.18^a^
Non-irradiated 0.009% GO encapsulated	100 ± 0.00^e^	100 ± 0.00^f^.	32 ± 1.11^d^
Irradiated 0.009% GO encapsulated	48.96 ± 0.88^c^	58.12 ± 0.79^c^	15.67 ± 0.23^b,c^
Non-irradiated 0.018% GO encapsulated	100 ± 0.00^e^	100 ± 0.00^f^.	32 ± 0.95^d^
Irradiated 0.018% GO encapsulated	50.41 ± 0.50^c^	60.66 ± 0.66^d^	16.13 ± 0.72^b,c^
Non-irradiated 0.045% GO encapsulated	100 ± 0.00^e^	100 ± 0.00^f^.	32 ± 0.70^d^
Irradiated 0.045% GO encapsulated	54.89 ± 0.91^d^	70.68 ± 0.61^e^	17.56 ± 0.51^c^
Non-irradiated 0.090% GO encapsulated	100 ± 0.00^e^	100 ± 0.00^f^.	32 ± 0.82^d^
Irradiated 0.090% GO encapsulated	47.21 ± 0.63^b^	56.38 ± 0.46^b^	15.11 ± 0.05^b^

The bioassay carried out on *E. kuehniella.*

*Note* The data are mean ± SE. Means within the same column, followed by a different letter are significant at p < 0.05, Duncan test. Spore count for treatments was carried out using three replicates. F = 153.214, df = 9, p = 0.0001. Mean is the average of three replicates 45 larvae per in treatment, F = 3069.403, df = 10, p = 0.0001.

**Figure 9:**
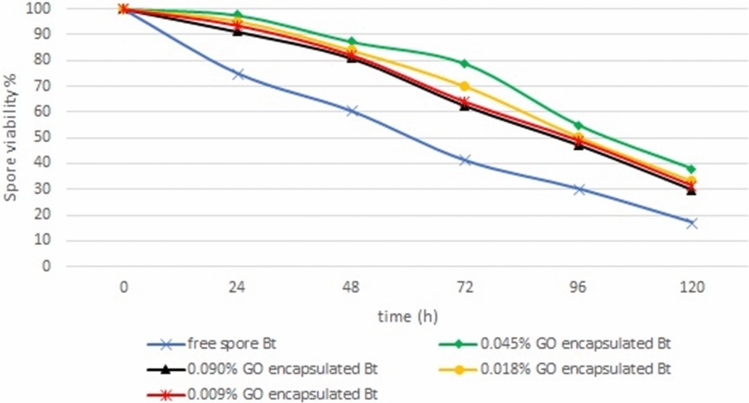
Effect of UV-A irradiation on spore viability of encapsulated *Bt* formulation with different concentrations of GO nanosheets for a different time exposure. The spore viability values represent the average of the three repetitions.

Figure 9 displays that after 96 h exposure to UV-A, spore viability decreased to 48.96, 50.41, 54.89, 47.21, 30.27% for the encapsulated formulation of *Bt* at different concentrations of 0.009, 0.018, 0.045, 0.090% w/v of GO, and free spore, respectively. As seen in Fig. 9, increasing GO nanosheets concentration in microencapsulated formulations from 0.009 to 0.045% w/v resulted in increased spore viability, while increasing the GO concentration up to 0.09% reduced it. However, this value generally fell for all formulations after 120 h UV-A exposure. During each exposure time, the *Bt* encapsulated formulations of 0.045% GO nanosheets were significantly higher than non-protected *Bt* formulation after UV-A exposure. So, this formulation provided the most photostable formulations.

The effect of UV-A on the *Bt* encapsulated formulation of GO nanosheets with different concentrations for 96 h was compared and the results are shown in Table 1. There were significant differences between all of irradiated *Bt* encapsulated and irradiated non-protected formulations, while there was no significant difference between 0.009% w/v GO formulation with 0.018% w/v GO formulation (p = 0.001). According to the obtained results of spore viability, among the four formulations of protected *Bt*, encapsulated formulation at a concentration of 0.045% has the highest protection of viability.

### Assessment of bioassay

**Figure 10:**
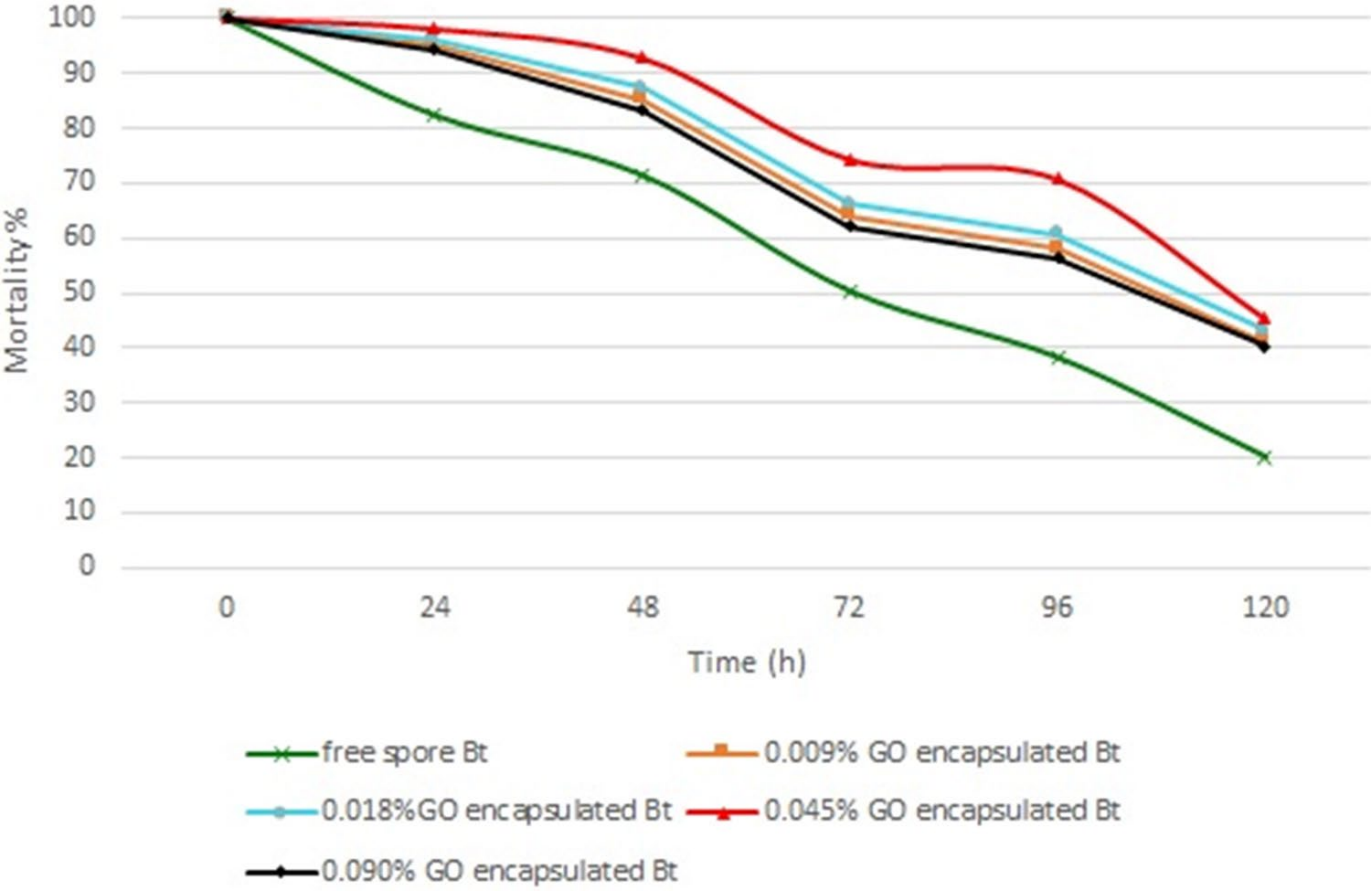
Effect of UV-A irradiation on mortality of larvae of encapsulated *Bt* formulation at different concentrations of GO for a different time exposure. The mortality values represent the average of the three repetitions.

Figure 10 shows the trend of mortality obtained in the laboratory for encapsulated *Bt* formulations with different concentrations of GO nanosheets during evaluated exposure times. Mortality evaluation against *E. kuehniella* larvae after 96 h of exposure to UV-A radiation showed that this factor declined to 58.12, 60.66, 70.68, 56.38, and 38.20% for the encapsulated formulations of *Bt* at different concentration of 0.009, 0.018, 0.045, 0.090% w/v of GO, and free spore, respectively. This is proportional to encapsulation efficacy of 51.68%, 54.61%, 66.17%, 49.67%, and 28.70%, respectively. According to the results of Table 1, there are significant differences between the four *Bt* formulations at p = 0.05. GO nanosheets at 0.045% w/v in *Bt* encapsulated formulation as a UV- protectants offered the highest UV protection.

## Discussion

The microencapsulated formulations of *Bt* were prepared by the Pickering emulsion technique using poly(methyl methacrylate-co-methacrylic acid) as latex particles. The reasons for choosing this polymer as the main constituent of colloidosomal microcapsules include:

- In environments with pH < 8 the polymer is water-insoluble and the cover of colloidosomal microcapsule is protected, while at pH > 8 (the pH of the midgut of *E. kuehniella*∼9) capsule release occurs and polymer dissolves in water. A 40 × magnification image of a polymeric capsule taken through an optical microscope illustrates the dissolution of the polymeric capsule wall at pH = 8.5 in about 14 s.
- According to the code of federal regulations title, 21 acrylic polymers are accepted for use in contact with food by the FDA^19^.
- Water was used as the main solvent and in the absence of any harmful organic solvent the emulsion polymerization reaction was performed.

NaCl was added to the aqueous phase to stabilize the emulsion against Ostwald ripening. GO nanosheets were added to the oil phase as a protective agent against UV. The UV absorption of GO has been shown by our previous researches^20^. Although the antibacterial activity of GO has been confirmed in some studies, the size of the nanosheets has been identified as an influencing factor on their antibacterial property. Therefore, larger GO nanosheets can display stronger antibacterial activity as compared to small GO nanosheets^21^. Olive oil was used in making the oil phase. A water/oil emulsion was obtained by mixing two phases. The mixing order was known as one of the main factors. Adding ethanol directly to the aqueous phase leads to aggregation of the latex before forming the shell. These results showed that the role of ethanol was to irreversibly fix the shell of the water-core emulsions and to stabilize the water-core emulsions. Therefore, using ethanol in the oil phase allowed the correct staging of events and successful colloidosome formation at low temperatures. This method does not involve high temperatures which is a necessary factor to maintain *Bt* bioactivity. Therefore, it is compatible with the encapsulation of microorganisms. According to research by Pusztai et al.^22^ the protein’s toxic effect is lost after 24 h of being exposed to the full solar spectrum. As reported by Poszgay et al., prolonged exposures up to 40 h resulted in the loss of spore count of *Bt*^23^*.* Hadapad et al.^24^ concluded that exposure of *Bt* to 96 h of UV radiations resulted in the loss of spore count. UV resistance results shown better efficiency of the sludge-based *Bt* formulations in comparison to the half-life of conventional *Bt* formulations ranging from 16 h to 2 days in field conditions^8^. In our previous study, we showed the highest mortality of irradiated combination formulation of GO/olive oil on second-instar larvae *E. Kuehniella* was 68.89%^20^. In this study, the same parameter for 96 h Irradiation of 0.045% GO encapsulated is 70.68%. Based on our previous study which showed that GO was a UV absorbent, during this study we checked their ability as a photo protectant after exposure to UV-A by the spore viability and mortality. Also, both experiments illustrated that the microencapsulation process confers extended bioactivity of *Bt* and that the microencapsulated *Bt* formulations are significantly more efficient than the free spore of *Bt*. Furthermore, the analysis of the results shows that the colloidosomal encapsulated formulations could be a promising and effective strategy for the extension of eco-friendly *Bt* insecticide.

## Materials and methods

### Materials

A known brand of Iranian olive oil was purchased from local markets in Kerman, Iran, and used as a continuous phase without purification. Graphite powder (< 300 µm, 99.99%) and ethanol (99.5%) were purchased from Sigma Aldrich (Steinheim, Germany). Methyl methacrylate, methacrylic acid, sodium dodecyl sulfate, ammonium persulfate, sodium chloride, potassium permanganate, hydrogen peroxide, hydrochloric acid, and sodium nitrate were purchased from Merck (Darmstadt, Germany). *B*. *thuringiensis* subsp. kurstaki KD-2 was provided by Green Biotech (Tehran, Iran).

### Synthesis of poly(methyl methacrylate-co-methacrylic acid) as latex particles

50 mL deionized water (DI), 10.65 g of methyl methacrylate, 4.24 g methacrylic acid, 0.20 g of sodium dodecyl sulfate as a stabilizer, and 0.12 g of ammonium persulfate as initiator were transferred to a 250 mL balloon flask and mixed and stirred at 350 rpm and degassed by bubbling nitrogen for 30 min. Then, the mixture was heated to 80 °C under a nitrogen blanket to avoid O_2_ introduction. After 10 h a latex was obtained. The particles were easily soluble in an alkaline environment (pH = 8).

### Synthesis of GO nanosheets

GO was synthesized according to a modified Hummer’s method. Briefly, 5.0 g of graphite powder, 115 mL of H_2_SO_4_, and 2.5 g of NaNO_3_ were added to a flask in an ice bath under vigorous stirring condition for 30 min. Then, it was followed by the slow addition of 15.0 g of KMnO_4_ for 2 h while maintaining the temperature at 35 °C. The reaction mixture was cooled to room temperature and 230 mL of DI water was added slowly to the solution. Eventually, the reaction was terminated by slowly adding 700 mL DI water and 30 mL H_2_O_2_. The final suspension turned bright yellow and was washed several times with HCl (3%, 80 mL) to remove the unreacted materials and then with DI water until neutrality. A homogenizer (Hielscher, UP400St, 20 kHz, 400 W, Germany) was used to the suspension in water laminated into GO nanosheets. Finally, the GO nanosheets were obtained in the form of a grey powder after freeze-drying^25^ for 72 h.

### Formation of the colloidosome

**Figure 11:**
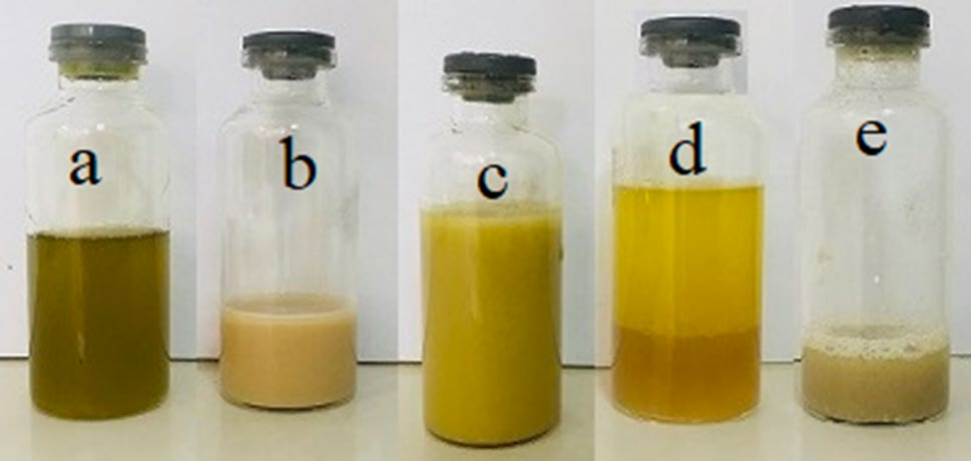
The preparation process of the formation of the colloidosome. (**a**) Olive oil + GO nanosheets (**b**) *Bt* + latex particles of poly(methyl methacrylate-co-methacrylic acid) + water. (**c**) Locking of the microcapsules upon addition of ethanol. (**d**) Microcapsules immediately after centrifugation. (**e**) Microcapsules after oil separation, that appear in the water.

The preparation process for the fabrication of water-in-oil (W/O) colloidosomes was as follows (Fig. 11). In a glass vial, 2 mL of ethanol was vortexed with 20 mL of commercial olive oil and 0.01 g of GO nanosheets for 1 min. In a separate glass vial, 1 g of dry powder of *Bt kurstaki* strain was added to 5 mL of the synthesized latex. Then, 5 mL of DI water containing 146 mg of NaCl was added and then vigorously vortexed for 1 min. After air bubbles disappeared from the mixture, the contents of both vials were added together. To form a stable emulsion, immediately the homogenization process of the mixture was performed with a vortex mixer for 1 min. The resulting colloidosomes were collected by repeated centrifugation at 4000 rpm, which separates colloidosomal microcapsules from the oil phase. Centrifugation was continued until no oil remained above the surface of the microcapsules.

### Characterization

X-ray diffraction (XRD) patterns were recorded on a Phillips X’Pert PRO (Panalytical, Netherlands) using filtered Cu Kα radiation (λ = 1.5417 Å) in the range of 2θ = 10°–80°. The morphology was investigated by scanning electron microscopy (FE-SEM, Sigma, Zeiss). For AFM analysis, dilution of different samples of each material was performed with water. A drop of the diluted suspension containing the microcapsules and some samples were placed on a lam and was allowed to dry in air. The plate was analyzed on a Veeco (Bruker/Veeco, Icon, USA). Tapping mode AFM was used for all images at 25 °C and analyzed with the Proscan software. The topography of materials was evaluated with AFM. Average roughness (*R*_a_) and root-mean-square (*Rms*) values were obtained for different samples. Transmission electron microscope (TEM) images were obtained by a LEO 912 AB transmission electron microscope (Zeiss, Germany).

### Ultraviolet resistance of encapsulated *Bt* formulations

The diluted encapsulated *Bt* formulation and nonprotected formulation of *Bt* with 10^8^ CFU mL^−1^as a control, were exposed to UV-A radiation in an open Petri dish at the same height and at 15 cm distance from the lamp (Philips, 15 W, white light 385 nm peak emission). To replace the water lost by evaporation; the volume of each sample was adjusted with sterile DI water before the determination of spore count. These formulations at different concentrations of GO (0.009, 0.018, 0.045, and 0.090%w/v) were subjected to UV-A treatment up to 120 h. The microcapsules were dissolved in a sodium hydroxide (pH = 8.5). Next, the released microcapsules were assessed for spore viability. The spore count of the samples was carried out by serial dilution with a nutrient agar medium (CFU). The percentage of spore viability was calculated by Eq. (1):

1 $${\text{Spore}}\;{\text{viability}}\;\left( \% \right) = \left[ {{{{\text{cfu}}_{{{\text{rad}}}} } \mathord{\left/ {\vphantom {{{\text{cfu}}_{{{\text{rad}}}} } {{\text{cfu}}_{0} }}} \right. \kern-\nulldelimiterspace} {{\text{cfu}}_{0} }}} \right] \times {1}00$$

where cfu_rad_ is the count of the irradiated spores (protected or nonprotected formulation), and cfu_0_ is the count of the initial non-irradiated spores (protected or nonprotected formulation. All the experiments were performed in triplicate and repeated on three different days.

### Bioassay procedures

Bioassays were carried out using second instars larvae of *E. kuehniella Zeller.* The larvae were reared using a mixture consisting of 1 kg flour and 0.5 kg of wheat bran as the nutrition source at 28 °C with 65% relative humidity. Fifteen larvae were selected and placed in a Petri dish, then 5 peanuts were immersed in various formulations as larval food, and dried for 5 min then added to Petri dishes. All procedures were performed under sterile conditions. These were incubated at 28 °C and 60% humidity. The percentage of mortality of larvae was recorded for all encapsulated formulations and compared to the control for 10 days. Each treatment was carried out in triplicate. The percentage of encapsulation efficacy was calculated by the Schneider Orelli equation (Eq. 2)

2 $${\text{Encapsulation}}\;{\text{efficacy}}\;\left( \% \right) = \left[ {\left( {X - Y} \right) \div \left( {100 - Y} \right)} \right] \times 100$$

where X is larval mortality percentage obtained in the treatment and Y is the mortality percentage in the negative control treatment (without *Bt* treatment).

### Statistical analysis

All results related to the determination of CFU counts, spore viability, and bioassays were the average of three replicates of three separate experiments. They were statistically analyzed by Statistical Package for the Social Sciences (SPSS) using the Duncan test performed after analysis of variance (ANOVA).
